# Parkinson's Disease Multidisciplinary Complex Therapy (PD-MCT): appreciation of its current function for the treatment of people with Parkinson’s disease in Germany and the needs of future development

**DOI:** 10.1007/s00702-025-02963-7

**Published:** 2025-06-12

**Authors:** Raphael Scherbaum, Matthias Höllerhage, Stephan Klebe, Peter Riederer, Thomas Müller, Nils Schröter, David Weise, Dirk Woitalla, Lars Tönges

**Affiliations:** 1 Department of Neurology, St. Josef-Hospital, Ruhr University Bochum, 44791 Bochum, Germany; 2 Department of Neurology, Hannover Medical School, 30625 Hannover, Germany; 3 Department of Neurology, Knappschaftskrankenhaus Recklinghausen, Recklinghausen, Germany; 4 Department of Neurology, Essen University Hospital, 45147 Essen, Germany; 5 Department of Psychiatry, Psychosomatics and Psychotherapy, Center of Mental Health, University Hospital Würzburg, 97080 Würzburg, Germany; 6 Department of Neurology, St. Joseph Hospital Berlin-Weissensee, 13088 Berlin, Germany; 7 Department of Neurology, Saarland University Medical Center, Homburg, Germany; 8 Department of Neurology, Asklepios Fachklinikum Stadtroda, Stadtroda, Germany; 9 Department of Neurology, University of Leipzig, Leipzig, Germany; 10 Department of Neurology, St. Josef-Hospital, Katholische Kliniken Ruhrhalbinsel, Contilia Gruppe, Essen, Germany

**Keywords:** Parkinson’s Disease, Parkinson’s Disease Multimodal Complex Therapy, Multidisciplinary, Physiotherapy, Occupational therapy

## Abstract

The inpatient Parkinson's Disease Multimodal Complex Therapy (PD-MCT) is a specialized therapeutic concept for people with Parkinson's disease (PD), that is increasingly applied with great success in Germany. It provides a core element of the provision of care for the more than 400,000 PD patients in Germany. In this expert review, we describe the basic elements of PD-MCT in Germany with its structural features, treatment team, therapeutic process and contents. The most recent data on the core neurologic value of PD-MCT are presented with its effect sizes and influencing factors. In detail, eight observational studies have demonstrated relevant effectiveness, with an average improvement of 7.8 points on the Movement Disorder Society Unified Parkinson’s Disease Rating Scale, Part III. Expert considerations of a PD-MCT target population are depicted as well as its additional values including multidisciplinary assessment and care, differential diagnosis and consideration of comorbidities, patient and caregiver education, improvement of nutrition, polypharmacy and therapeutic drug monitoring. The review concludes with a viewpoint on the possibilities for optimization and the future needs for PD-MCT.

## Introduction

Due to the complex spectra of intra- and interindividual clinical manifestations of Parkinson’s Disease (PD), a person-centered, individualized, and multidisciplinary approach is essential. Multidisciplinary care improves symptoms, daily activities, quality of life, and reduces costs (Lidstone et al. 2020). On an individual level, this applies to physiotherapy (PT) (Ernst et al. 2024; Ypinga et al. 2018), occupational therapy (OT) (Tofani et al. 2020, Sturkenboom et al. 2014), or speech and language therapy (SLT) (Pu et al. 2021; Sackley et al. 2024; Maas et al. 2024), across outpatient (van der Kolk et al. 2019), inpatient (Steendam-Oldekamp and van Laar 2024; Monticone et al. 2015), and integrated (Rajan et al. 2020) settings. However, many people with PD tend to be underserved with multidisciplinary care in the outpatient or community-based sector of the hospital-focused German healthcare system (Heinzel et al. 2018). Up to 42% of the 420,000 people with PD receive only medication, with only 36%, 6%, and 4% receiving PT, OT, and SLT, respectively (Heinzel et al. 2018). Thus, multidisciplinary care is mainly provided in the inpatient sector. PD Multimodal Complex Therapy (PD-MCT) accounts for about a quarter of inpatient stays for movement disorders, with increasing case numbers (Richter et al. 2019; Scherbaum and Tönges 2024). In 2022, PD-MCT was applied over 15,000 times (Scherbaum and Tönges 2024). During a one- to three-week hospitalization, symptoms and daily functions are improved through pharmacological and non-pharmacological strategies. While previous reviews focused on different or more general settings of multidisciplinary care in PD, inpatient multidisciplinary care in PD was only recently reviewed (Steendam-Oldekamp and van Laar 2024). This article discusses the features, value, target group, additional benefits, and future aspects of PD-MCT as a specific type of inpatient multidisciplinary PD care in Germany.

## What is Parkinson’s Disease Multimodal Complex Therapy?

### Structural features

PD-MCT is a multidisciplinary inpatient therapy aimed at optimizing functional ability, reducing disability, and promoting quality of life. Defined by the German reimbursement system, it follows the German modification of the International Classification of Procedures in Medicine (ICPM), the Operation and Procedure Classification System [OPS, code 8-97d (Federal Institute for Drugs and Medical Devices 2024)]. PD-MCT requires 7.5 h of non-pharmacological therapies per week (thereof 5 h as individual treatment), weekly team meetings under neurological supervision, and documentation of treatment goals and results. It can be applied for 7–13, 14–20, or more than 21 days (Federal Institute for Drugs and Medical Devices 2024), with most PD-MCTs lasting 14–20 days (Richter et al. 2019). Conducted in over 200 (Richter et al. 2019) hospitals in Germany, its use is increasing. The terms ‘multimodal’ and ‘complex’ highlight its multidisciplinary nature, involving collaboration among various professionals, however, without a complete synthesis of approaches (Rajan et al. 2020). PD-MCT was introduced in 2008 to adjust the reimbursement structures for hospitals that had seen high costs for complex and long hospital stays after the introduction of a DRG system in Germany, which increased the risk of inadequate care.

### Therapeutic team

**Fig. 1:**
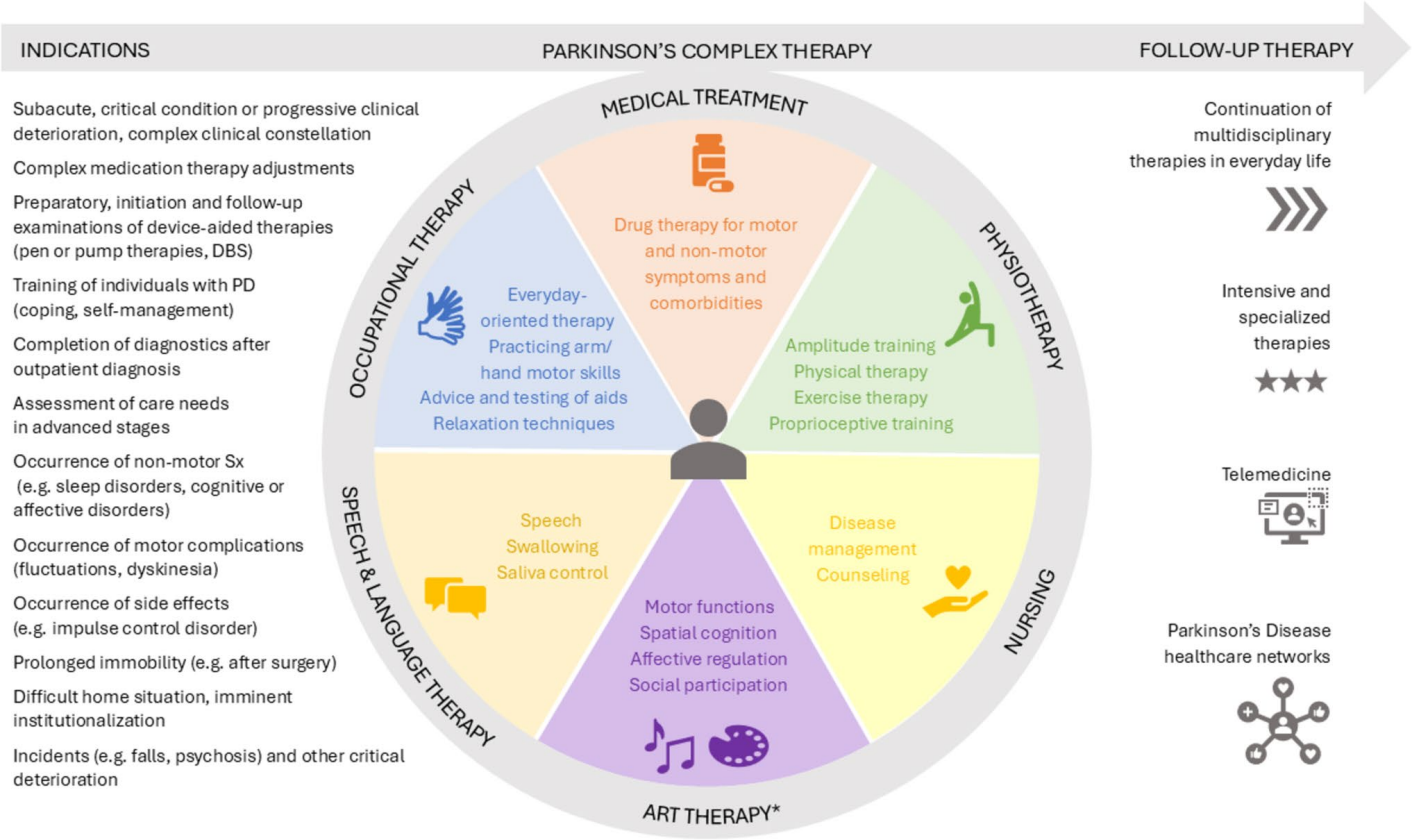
Indications, contents, and follow-up therapies of PD-MCT. *DBS* deep brain stimulation, *Sx* symptoms. *As an example, further additional therapies include physical, sports, music, or psychotherapy

In PD-MCT, pharmacological therapy is complemented by non-pharmacological approaches, including PT, OT, SLT, physical, sports, art, music, or psychotherapy (Federal Institute for Drugs and Medical Devices 2024), depending on the center’s resources (Fig. 1). Reimbursement requires involvement from at least three different healthcare professions (Federal Institute for Drugs and Medical Devices 2024), in addition to regular nursing staff and neurologists. PT or OT are mandatory, while other therapies may vary and are optional from a formal viewpoint (Federal Institute for Drugs and Medical Devices 2024). The team can additionally include PD nurses, neuropsychologists, social workers, urologists, gastroenterologists, and nutritionists, based on local availability and clinical needs (Qamar et al. 2017). In the sense of person-centered medicine, people with PD and their caregivers are involved as active team members (self-management), guiding care based on their values and preferences, and supporting their health and life goals (The American Geriatrics Society Expert Panel on Person‐Centered Care 2016).

### Process

Before the inpatient stay, an outpatient consultation assesses the indication and suitability for PD-MCT and sets therapy goals with the person with PD.

Upon admission, each healthcare professional evaluates the condition of the individual with PD and identifies core problems. Individual therapy goals are set by the team in consultation with the affected and adjusted as needed. So far, there is no standardization of goals establishment using methods such as Goal Attainment Scaling (Kiresuk and Sherman 1968). Then, targeted interventions including drug treatment are provided with tailored content, frequency, and intensity. PD-MCT follows an individualized healthcare approach, considering clinical subtypes, genetics, personality, lifestyle, aging, and comorbidities (Titova and Chaudhuri 2017b). Interventions are tailored to the varying needs throughout the course of the disease (Weise et al. 2024).

At the end of therapy, final discussions are held with the person with PD and their relatives to ensure the transfer of learned strategies and exercises into daily life. A doctor’s letter summarizes the diagnosis, treatment, and recommendations for the general practitioner and community neurologist. Currently, there usually is no direct communication between PD-MCT centers and community therapists but novel communication approaches are currently being evaluated (Naegele et al. 2024).

### Contents

Neurologists handle therapy coordination, history taking, clinical exams, diagnostics, differential diagnosis of new issues, and drug treatment. Possible diagnostic tools include the MDS PD diagnostic criteria (Postuma et al. 2015), neuropsychological tests, and Flexible Endoscopic Evaluation of the Swallowing Act (FEES). Medical treatment follows the PD guideline of the German Society of Neurology (Höglinger and Trenkwalder et al. 2023) and current scientific recommendations (Fox et al. 2018; Seppi et al. 2019). Frequent focus areas include complex medication adjustments and start or follow-up examinations of device-aided therapies (pen or pump therapies, deep brain stimulation).

PT for PD aims to improve physical capacity, transfers, manual activities, balance and falls, and gait, as outlined in the European PT Guideline for PD (Domingos et al. 2018). Techniques include amplitude training (LSVT-BIG), proprioceptive training, everyday-oriented therapy, medical training therapy, and home program instructions. Supplementary therapies involve massage and heat applications. Effective interventions for gait disorders include 95 compensatory strategies, such as walking at the rhythm of a metronome or raising knees high (Nonnekes et al. 2019). Specialized PD PT reduces complications, mortality, and costs compared to conventional PT (Ypinga et al. 2018). Higher doses of physical training seem to improve motor function and quality of life stronger than lower doses (Schenkman et al. 2018; Rafferty et al. 2017).

OT aims to improve daily functions, maintain social roles, enhance autonomy, and compensate for limitations. The Guidelines for OT in PD Rehabilitation (Sturkenboom et al. 2011) propose ten intervention areas: self-management, daily structure, stress management, arm/hand motor skills, focused attention, cognitive movement strategies, minimizing dual tasks, using cues, optimizing the physical environment, and caregiver advice. Everyday-oriented therapy and compensation strategies, such as for eating or personal hygiene, play a major role. OT overlaps with PT in areas like fall prevention and amplitude training. It improves the quality of life, independence, and mobility in people with PD (Tofani et al. 2020, Sturkenboom et al. 2014).

SLT focuses on communication and swallowing. Detailed speech exams lead to therapies targeting vocal volume, pitch range, speech intelligibility, and rate. Swallowing training aims to improve effectiveness and safety. Diagnostics and follow-up use questionnaires, clinical exams, and technical procedures like FEES. Expiratory Muscle Strength Training and dopaminergic medication benefit dysphagia (Claus et al. 2021). The European Guideline for SLT in PD (Kalf et al. 2011) suggests modifying food volume and consistency and speech amplitude training [short components from the usually four-week LSVT-LOUD protocol (Sackley et al. 2024)].

## What is the core (neurologic) value of Parkinson’s Disease Multimodal Complex Therapy?

### Effect sizes from previous studies

To date, eight observational studies on the effectiveness of PD-MCT have been conducted with 1246 patients at seven centers, specifically relating to this German construct (Brücher et al. 2019; Decher et al. 2023; Heimrich and Prell 2021; Müller et al. 2017; Scherbaum et al. 2020, 2022; Wagner et al. 2022; Ziegler et al. 2022). These studies showed positive short-term effects of PD-MCT on quality of life, everyday functions, non-motor and motor symptoms, and sensor-based gait parameters for the average sample. For instance, motor symptoms improvement was shown in five studies with an average of 7.8 points on the MDS-UPDRS III (ranging from 4.7 to 12 points) (Brücher et al. 2019; Heimrich and Prell 2021; Scherbaum et al. 2022; Wagner et al. 2022). However, these studies had heterogeneous outcomes, lacked control groups, and often did not include long-term follow-up (Brücher et al. 2019; Decher et al. 2023; Scherbaum et al. 2022; Müller et al. 2017). As an example, one observational study with 47 mildly to moderately affected patients showed immediate improvements in motor symptoms, quality of life, and depression, with only motor symptoms remaining improved after six weeks (Scherbaum et al. 2020). Motor symptoms were reduced by an average of 4.7 points on the MDS-UPDRS III at discharge, roughly equivalent to annual progression rates. The gain in quality of life through PD-MCT was comparable to the loss in quality of life over 2 years. Another monocentric study of 591 cases showed an improvement of 2.4 points in everyday motor function on the MDS-UPDRS II (Ziegler et al. 2022).

In general, inpatient multidisciplinary therapies improved motor symptoms, everyday functions, and quality of life in four international randomized controlled trials (Monticone et al. 2015; Ferrazzoli et al. 2018; Marumoto et al. 2019; Frazzitta et al. 2012). Some effects lasted up to one year post-intervention. However, these concepts are only partially comparable to PD-MCT, as they involve more intensive and longer programs (12–21 therapy hours/week over 4–8 weeks). The clinical endpoints, content, intensity, duration, and follow-up data were also heterogeneous.

Currently, there is no such higher-level evidence on the effectiveness of PD-MCT although widely used in Germany. In addition, the share each component (drugs, therapies, placebo) contributes to the outcome has not been determined, e.g. by modeling the outcome as a function of Levodopa-equivalent daily dose, the time spent with PT/OT/SLT sessions, or sensor-based physical activity during PD-MCT. A pragmatic randomized controlled trial on the clinical and biological effectiveness of PD-MCT compared to standard outpatient therapy has been recruiting participants since October 2023. This trial includes comprehensive clinical, patient-related, and biological endpoints, as well as digital biomarkers of gait and mobility in the home setting as exploratory endpoints (DRKS00032619).

### Influencing factors

PD-MCT appears to be particularly suitable for distinct subgroups of individuals with PD in terms of achieving short-term therapeutic success. Identifying the target group is crucial for the efficient allocation of scarce healthcare resources and the appropriate application of PD-MCT. At the same time, scientific statements on predictors of PD-MCT efficacy are derived from group-related data in monocentric observational studies (Heimrich and Prell 2021; Scherbaum et al. 2020; Ziegler et al. 2022). Individual treatment goals, the varying weighting of clinical endpoints, and the expertise of specialists necessitate case-based consideration when determining the indication for PD-MCT. Hence, the quality and quantity of therapy success depends on individually set goals and outcomes meaningful to individuals with PD.

Evidence suggests that a high burden of motor symptoms, a low burden of depressive symptoms, and younger age increase the likelihood of motor improvement (Heimrich and Prell 2021; Scherbaum et al. 2020). For everyday functions, their greater impairment at the start of PD-MCT, younger age, good fine motor skills, absence of psychiatric or cognitive disorders, and low step time variability are associated with better outcomes (Ziegler et al. 2022; Oppermann et al. 2024). These findings indicate that individuals in the middle of the severity spectrum have a particularly good chance of benefiting from PD-MCT in terms of motor symptoms and everyday functions. Ultimately, treatment outcomes must be measured against the defined treatment goals for each case. Future studies could use methods such as Goal Attainment Scaling (Kiresuk and Sherman 1968) to further define and elucidate therapy success.

There is a lack of data from randomized controlled trials with longer follow-up periods on the factors influencing positive PD-MCT effects. Additionally, the roles of behavioral factors such as treatment adherence, use of health services, and daily physical activity after discharge in short- and long-term outcomes remain unclear. Further research is needed to elucidate these aspects and optimize the use of PD-MCT in PD management. As centers vary in terms of the number of cases per week, ranging from more than two (25 centers covering almost 60% of cases) to less than one (75% of centers covering just over 25% of cases) (Richter et al. 2019), system-level factors such as center characteristics or socioeconomic factors may also influence outcomes. Future studies may adopt mixed-methods designs including interviews with users, providers, and payers as well as multi-site collection of individual participant data or secondary data from claims-based analyses using proxies of effectiveness, e.g. fractures from falls or re-hospitalization rates (Ypinga et al. 2018), to determine the conditions of utilization, effectiveness and improvements thereof and ultimately develop recommendations on provision and use of PD-MCT.

## The target population of Parkinson’s Disease Multimodal Complex Therapy

The goals of PD-MCT vary and take individual factors into account. These include the patient's age, life circumstances (e.g. ability to work), and, in particular, the specific characteristics of the illness.

In young patients, a therapy should be determined that, in addition to symptom reduction, enables long-term adjustment while minimizing therapy-associated complications. The importance of the expertise of treatment units specializing in PD should be emphasized here. Another important therapeutic goal is to educate patients about the progression and variety of symptoms that characterize the disease, as well as the importance of regular physical exercise, which should ideally be practiced in the professional setting of a PD-MCT.

The treatment of elderly patients often pursues other goals. These include maintaining physical autonomy, providing suitable aids, and managing further outpatient care. Intensified exercise treatment in combination with the readjustment of medication forms an important basis for this. In particular, the added value of daily exercise treatment in an inpatient setting compared to low-frequency therapy in an outpatient setting should be emphasized, which has been proven to lead to functional improvement through PD-MCT treatment (Scherbaum et al. 2020).

PD patients with motor fluctuations represent a particular challenge for therapy. A precise analysis of the fluctuations is a prerequisite for optimizing drug therapy and can only be achieved through continuous monitoring and modification of the medication. Numerous questionnaires are available for self-assessment, but a detailed analysis is only possible by comparing the subjectively perceived mobility with a medical evaluation. Continuous observation in an inpatient setting as part of PD-MCT is therefore particularly valuable for this patient group. The entire team of therapists can jointly assess and evaluate the various facets of the motor complication.

The same applies to patients with DBS and a need to reprogram. Often the mobility only changes after reprogramming with a considerable time latency, so the outpatient readjustment is rarely followed by success.

The treatment of so-called levodopa unresponsive symptoms (Sethi 2008) can only be solved by a joint therapeutic effort by a specialized team. Drug therapies do not help in all cases; the importance of physiotherapeutic strategies must be emphasized in these patients and, after an initial analysis, requires the rehearsal of various coping strategies, which must be continuously reevaluated for their effectiveness.

In addition to the medical indications and aspects mentioned, PD-MCT has an unquantifiable relevance for the basic attitude of patients in dealing and coping with the disease. Similar to psychotherapeutic procedures, the patient does not feel alone in the treatment setting with other patients and experiences significant support in coping with the burden of PD.

## What are the additional values of Parkinson’s Disease Multimodal Complex Therapy

### Multidisciplinary assessment and care

Multidisciplinary care needs to correctly assess and manage individual motor and non-motor symptoms (Lidstone et al. 2020; Weise et al. 2024). The members of a multiprofessional team such as movement disorder specialists, physical and occupational therapists, speech-language therapists, neuropsychologists and PD nurses evaluate patients during the entire PD-MCT stay at different time points in different motor and non-motor states. While outpatient consultations rely on patient-reported data, PD-MCT offers continuous assessment throughout the day. This comprehensive monitoring allows for optimized pharmacological adjustments, including the management of nocturnal symptoms (Stefani and Högl 2020). Hence, such multidisciplinary evaluation in a team setting results in a broader overview of individual patients’ deficits and consequently in a more tailored individual therapy (Ziegler et al. 2022). Furthermore, other disciplines and specialists such as dietitians, social workers, urologists, or cardiovascular specialists can be involved in this purpose (Radder et al. 2019; Weise et al. 2024).

### Differential diagnosis and consideration of comorbidities

The differential diagnosis of Parkinsonian syndromes can be challenging both at the onset and during the course of the disease, especially since atypical Parkinsonian syndromes may have prolonged progression and may only become apparent as such over time. PD itself is heterogeneous in the clinical presentation, disease progression, burden of non-motor symptoms, and response to dopaminergic therapy (Fereshtehnejad et al. 2017). A thorough, multi-stage, interdisciplinary assessment within the framework of PD-MCT may support accurate diagnostic classification, prognosis estimation, and therapeutic decision-making. Moreover, PD is characterized by significant comorbidities, particularly neuropsychiatric conditions such as depression, dementia, or psychosis, as well as sleep and swallowing disorders, pain, and internal medical comorbidities (Schapira et al. 2017; Csoti et al. 2016), all of which require specialized diagnostics (e.g. FEES (Vogel et al. 2022), autonomic testing (Leys et al. 2020) or neuropsychological testing) and treatment.

### Optimization of therapy in an advanced stage

In the advanced stages of PD, treatment becomes increasingly complex, requiring a combination of multiple oral medications or non-oral follow-up therapies. Pharmacological management must be tailored to each patient’s specific needs (Armstrong and Okun 2020). Moreover, non-oral PD therapies for advanced stages, such as DBS and medication pumps, require extensive individual adaptation. Optimization of DBS settings can be time-consuming (Koeglsperger et al. 2019). PD-MCT provides the opportunity to test various DBS settings and observe their effects over hours or days, reducing the risk of discharging a patient with suboptimal settings. Similarly, infusion therapies such as levodopa/carbidopa intestinal gel (LCIG), levodopa/carbidopa/entacapone intestinal gel (LECIG), or continuous subcutaneous administration of foslevodopa/foscarbidopa or apomorphine require dose optimization (van Laar et al. 2023), which is more feasible during PD-MCT. This includes the possibility for nasogastric test periods for LCIG/LECIG therapy, allowing patients to evaluate treatment effects before committing to percutaneous endoscopic gastrostomy (PEG) placement, thereby making a well-informed decision.

### Patient and caregiver education

Due to the complexity of PD, which involves both motor and non-motor symptoms (Schapira et al. 2017) as well as comorbidities (Csoti et al. 2016), distinguishing between PD-related symptoms and those arising from other conditions can be challenging. Misattributing symptoms to PD can lead to unrealistic expectations about medication efficacy and subsequent disappointment. Advanced-stage patients may require complex medication regimens or non-oral therapies. These advanced non-oral treatment options, including DBS and pump therapies, necessitate thorough patient and caregiver education regarding handling but also expected benefits and limitations to ensure realistic expectations (Mameli et al. 2023; Fung et al. 2024). However, outpatient settings often lack the time necessary for comprehensive education. PD-MCT provides a unique opportunity to thoroughly inform patients and caregivers about the full spectrum of PD symptoms, including non-motor aspects, comorbidities, and the expected responses to different treatment options. This structured education improves treatment adherence and facilitates more realistic expectations regarding disease progression and therapy outcomes.

### Improvement of nutrition

Levodopa’s pharmacokinetic properties significantly depend on nutritional factors because levodopa competes for intestinal absorption and brain uptake with other large neutral amino acids (LNAA) including, e.g., phenylalanine, tyrosine, valine, and leucine (Cedarbaum 1987; van Kessel and El Aidy 2019) from meals rich in proteins (Leta et al. 2023; Müller et al. 2024). Besides these drug-nutrient interactions, factors influencing the clinical benefit of levodopa include delayed gastric emptying (Nyholm and Lennernäs 2008), Helicobacter pylori infection, gastric acidity, the composition of gut bacteria, and gastrointestinal disorders (Müller et al. 2024). To reduce the loss of levodopa across the various cellular uptakes, its combined application with a catechol-*O*-methyltransferase inhibitor, like entacapone or opicapone, as well as subcutaneous and intestinal infusions are suitable. Due to its duration and multidisciplinary setting including dieticians, PD-MCT offers an opportunity to design nutritional interventions that optimize levodopa pharmacokinetics, improve gastrointestinal dysfunction, and minimize protein interactions (Müller et al. 2024).

### Evaluation of polypharmacy

In PD, polypharmacy is a frequent problem, as drugs other than those prescribed to ameliorate PD symptoms belong to the standard therapeutic approaches. Reasons for multi-drug regimens include the advanced age of patients with PD who often additionally suffer from other age-related diseases/disorders, like diabetes type II that is strictly associated with PD (Iuliis et al. 2022), and cardiovascular dysfunctions/insufficiencies, rheumatic disorders, dysfunctions of the lipid metabolism, kidney problems, or other health problems (Müller et al. 2024). Antihypertensive medications, e.g., β-blockers and diuretics are among the first-line medications of arterial hypertension; anti-depressive drugs with anticholinergic properties may delay gastric emptying and thus reduce the benefit of levodopa and selective inhibitors of monoamine oxidase B (selegiline, rasagiline, safinamide) should not be administered together with selective serotonin reuptake inhibitors to avoid the serotonin syndrome. PD frequently is associated with diabetes type II and antidiabetic drugs, like metformin and thiazolidinediones and eventually intranasal insulin are prescribed in PD. To antagonize enhanced plasma concentrations of cholesterol cholesterol-lowering drugs are used in PD to improve cognitive abilities. Antirheumatic drugs like chloroquine/hydroxychloroquine seem to reduce the risk for PD (Müller et al. 2024). PD-MCT provides sufficient time for a critical appraisal of multi-drug regimens.

### Therapeutic drug monitoring

Despite a limited level of recommendation, using therapeutic drug monitoring (TDM) allows for the levodopa treatment to be individualized and further optimized by analyzing the plasma concentrations, especially of levodopa on an individual basis (Hiemke et al. 2018; Müller et al. 2024).

TDM has not yet been established for clinical practice in patients with PD. To enhance therapeutic efficacy and tolerability, the determination of plasma levels of PD drugs recently gained more and more importance in the development of substances with an improved pharmacokinetic profile. Typical examples are extended-release formulations of dopamine agonists, add-on of amantadine and new intrajejunal or subcutaneous as well as inhalation formulations of levodopa particularly (Modi et al. 2019a, 2019b; Marmol et al. 2021; LeWitt et al. 2022; Rosebraugh et al. 2021; Danysz et al. 2021; Riederer et al. 2025). TDM is an essential step to optimizing drug treatment on an individualized, personalized basis. It may reduce the risk for drug-drug interactions substantially, control dose regimes, prove compliance, contribute to clarifying non-response at therapeutic doses, and improve drug adherence and suboptimal tolerability (Müller et al. 2024; Titova and Chaudhuri 2017a, b; Riederer et al. 2025). For example, determinations of plasma levodopa in a TDM setting may gain more importance in the near future. Calculation of levodopa plasma levels may allow clinicians to differentiate better between branded and generic levodopa formulations. In the past years, the impact of health care authorities, politicians, and payers rose in terms of drug cost reduction. Various approaches of non-transparent price regulation scenarios of country-specific healthcare systems increasingly influence drug selection and accordingly limit therapeutic options. Generally, this system supports the prescription of cheaper, generic available drugs, however, these compounds considerably vary in their pharmacokinetic behavior. In Germany, even the pharmacist may switch between generic and branded compounds without information for the prescribing neurologists (Riederer et al. 2025). Therefore, the efficacy of a personalized PD drug treatment cocktail may change, particularly when compounds with a short half-life are applied. Therefore, the effects of oral levodopa formulations are particularly altered by differences between the original and the various generic available oral levodopa formulations. This scenario is due to country-specific, sometimes even regionally different, non-transparent drug price reduction- and discount procedures. It is supplemented by direct or hidden modes of budget limitations for prescribing neurologists, i.e. in Germany. Therefore, TDM for the various levodopa formulations in a defined, strictly standardized fashion will particularly gain importance in the future to counteract this currently ongoing development, which may provide harm to patients even in terms of drug safety.

Generally, TDM in PD has not been established, so far. TDM will help clinicians determine the dosing and the efficacy of an applied PD drug combination in relation to the clinical response, predominantly motor behavior. For instance, among others, important influencing factors are altered gastrointestinal motility by dopamine substitution, body weight, respectively the body mass index or progression of PD (Valente et al. 2024; Woitalla et al. 2006). Therefore, TDM is a matter of future development. It will help to exclude absorption problems, determine metabolism rates, and as a tool to detect compliance problems. TDM in PD patients has the potential to become an important tool for individualized assessment of levodopa plasma bioavailability in particular for clinical practice. As aforementioned TDM will help to measure the as till underestimated diversity between the original "branded" and the various generic PD drug formulations. This will perhaps convince authorities, health politicians, and financial services that various PD drug preparations, containing the same amount of active ingredients, should not indiscriminately be exchanged for pricing reasons.

## Optimization and future needs for Parkinson’s Disease Multimodal Complex Therapy

Despite the success of PD-MCT in improving patient outcomes over the past decade, there remain opportunities for further optimization to ensure it meets future needs. Key areas for improvement include education, expansion, and adoption for patients with atypical Parkinsonian syndromes, the integration into PD networks to improve the sustainability of the improvements of the individual patients, and the integration of new technologies.

Education plays a central role in the advancement of PD-MCT for PD. Patients and their caregivers should be educated about both the motor and non-motor symptoms of PD. This knowledge can lead to a better understanding of symptoms that often lead to a significant reduction in quality of life, such as depression, impulse control disorders, nocturia, and visual hallucinations. Recognizing these symptoms as part of PD allows for goal-directed communication with healthcare professionals, reduces the stigma associated with these symptoms, facilitates treatment, and ultimately improves quality of life.

In recent years, it has become increasingly evident that lifestyle changes can have a lasting positive impact on the course of the disease (Reichmann et al. 2022; Ernst et al. 2024). Many of these changes can be made independently following a structured analysis of the patient's individual risk profile. In addition, the involvement of the patient's social environment can greatly enhance the success of these interventions. As part of PD-MCT, patients should also be educated about continuous therapeutic approaches, such as pump-based therapies or DBS. Early education about these options can facilitate access to these therapies and ensure that patients are transitioned to these continuous therapies on time. To maintain the symptomatic improvements achieved during PD-MCT in the long term, therapies initiated in the inpatient setting should be continued in the outpatient setting by experienced and specially trained therapists. This requires close links between inpatient and outpatient services, for example through PD networks or telemedicine approaches as promising tools for suitable PD patients (Cubo and Delgado-López 2022). Such integration is essential to ensure that the effects of PD-MCT are not short-lived. Further research on the PD-MCT target group and factors enabling long-term effectiveness is crucial for the efficient allocation of scarce healthcare resources given the oftentimes short-lived effects of multidisciplinary care.

Another promising area is the use of digital health applications. Introducing these tools during inpatient treatment allows patients to become familiar with them early on, making it easier to integrate them into everyday life. Home-based exercise training, for example via mobile apps, could be a promising way to maintain the improvement of PD-MCT (Putzolu et al. 2023). Another potential application includes app-based support for dietary changes, such as switching to a Mediterranean diet.

Finally, improved expectation management is essential as already shown for other treatment options in PD like DBS (Mameli et al. 2023). Clearly defining and documenting realistic treatment goals can significantly improve patient satisfaction by aligning expectations with achievable outcomes.

In addition, the success of PD-MCT, as demonstrated in numerous studies, should at best be extended to the treatment of patients with atypical Parkinsonian syndromes, such as Lewy body disease, corticobasal syndrome, progressive supranuclear palsy, or multiple system atrophy. However, it is important to recognize that the PD-MCT concept cannot be simply applied to all these conditions in a similar fashion but should be tailored to their specific needs. Given the rapid progression of these disorders, psychological support and assessment of the home environment should be an integral part of therapy. This approach would help identify potential support needs at an early stage and facilitate access to the necessary resources, ensuring that patients and caregivers have the support they need to manage these complex conditions effectively.

## Outlook

The introduction of PD-MCT was a success story for the treatment of PD patients in Germany, given the internationally rather limited availability of PD inpatient care. The continuous improvement and further development of the applied therapeutic measures as well as the expertise and knowledge of the specialists offering the treatment are impressive. Important aspects for the future are the development of patient education, the seamless integration of PD-MCT into patient treatment networks, the expansion to atypical Parkinsonian syndromes, and the integration of digital tools for communication, symptom control, and, at best, therapy feedback and optimization. The current, increasingly difficult overall situation in the healthcare system leading to ‘outpatientization’ will pose major hurdles to the further development of PD-MCT. Therefore, every single aspect of the PD-MCT procedure should be constantly and carefully re-evaluated to identify the potential to improve their efficient application.

## Data Availability

The data included in this review are taken from cited literature.
